# Supplementary material for: Look at My Body: It Tells of Suffering—Understanding Psychiatric Pathology in Patients Who Suffer from Headaches, Restrictive Eating Disorders, or Non-Suicidal Self-Injuries (NSSIs)
Source: Pediatr Rep. 2025 Feb 8;17(1):21. doi: 10.3390/pediatric17010021 (PMC11858191; doi:10.3390/pediatric17010021)
Supplement: Supplementary file 1 [file pediatrrep-17-00021-s001.zip › Table S2.pdf]

Table S2. Comparisons of psychodiagnostic instruments in the three groups and post hoc analyses corrected for the Bonferroni test.

**Variables**		**Headaches** N=20		**REDs** N=20		**NSSIs** N=20					**a—b**	**a—c**	**b—c**	**Contrast**
		M	SD	M	SD	M	SD	F	*p*	ω ²	*p*	*p*	*p*	
K-SADS-PL	Depression	0.500	0.513	0.850	0.366	1.000	0.000	9.940	**<.001*****	.230	**.023***	**<.001*****	0.761	a<b; a<c
	Mania	0.000	0.000	0.100	0.308	0.200	0.410	2.280	0.112	.041	-	-	-	-
	Hypomania	0.050	0.224	0.150	0.366	0.150	0.366	0.628	0.537	-.013	-	-	-	-
	Dysregulation	0.200	0.410	0.050	0.224	0.050	0.224	1.676	0.196	.022	-	-	-	-
	Psychosis	0.100	0.308	0.350	0.489	0.450	0.510	3.279	**.045***	.071	0.261	**.050***	1.000	a<c
	Panic	0.450	0.510	0.550	0.510	0.550	0.510	0.256	0.775	-.025	-	-	-	-
	Agoraphobia	0.250	0.444	0.200	0.410	0.100	0.308	0.760	0.472	-.008	-	-	-	-
	Separation anxiety	0.400	0.503	0.450	0.510	0.100	0.308	3.537	.036*	.078	1.000	0.129	0.055	-
	Social anxiety	0.150	0.366	0.400	0.503	0.650	0.489	5.987	**.004****	.143	0.329	**.004****	0.329	a<c
	Phobia	0.300	0.470	0.400	0.503	0.150	0.366	1.563	0.218	.018	-	-	-	-
	GAD	0.500	0.513	0.500	0.513	0.400	0.503	0.257	0.774	-.025	-	-	-	-
	OCD	0.350	0.489	0.450	0.510	0.100	0.308	3.279	**.045***	.071	1.000	0.261	**.050***	b>c
	AN	0.100	0.308	0.900	0.308	0.300	0.470	25.333	**<.001*****	.448	**<.001*****	0.206	**<.001*****	a<b; b>c
	ADHD	0.100	0.308	0.050	0.224	0.050	0.224	0.257	0.774	-.025	-	-	-	-
	ODD	0.050	0.224	0.050	0.224	0.100	0.308	0.257	0.774	-.025	-	-	-	-
	Conduct disorder	0.000	0.000	0.050	0.224	0.100	0.308	1.036	0.361	.001	-	-	-	-
	PTSD	0.200	0.410	0.158	0.375	0.100	0.308	0.375	0.689	-.022	-	-	-	-
IQ	Tot	109.25	14.65	111.89	13.05	103.05	18.57	1.655	0.200	.022	-	-	-	-
	VCI	111.60	12.33	113.37	14.31	106.45	16.04	1.242	0.297	.008	-	-	-	-
	PRI	108.90	19.05	114.00	13.68	108.65	16.39	0.642	0.53	-.012	-	-	-	-
	WMI	101.65	12.95	95.73	12.08	91.15	20.61	8.117	**.0172***	.040	0.582	**.013***	0.392	a<c
	PSI	103.55	18.49	106.89	18.14	98.35	20.29	1.004	0.373	.000	-	-	-	-
SCID-5-PD[a]	Avoidant	0.154	0.376	0.353	0.702	0.474	0.697	3.097	0.542	.000	-	-	-	-
	Dependent	0.000	0.000	0.235	0.562	0.000	0.000	6.015	0.198	.035	-	-	-	-

Obsessive-Compulsive	0.308	0.480	0.529	0.800	0.316	0.749	8.021	0.908	-.020	-	-	-	-
Paranoid	0.077	0.277	0.000	0.000	0.053	0.229	1.224	0.542	-.017	-	-	-	-
Schizotypal	0.154	0.376	0.188	0.332	0.105	0.315	0.175	0.916	-.039	-	-	-	-
Schizoid	0.000	0.000	0.000	0.000	0.053	0.229	1.612	0.447	-.009	-	-	-	-
Histrionic	0.000	0.000	0.000	0.000	0.053	0.229	1.612	0.447	-.009	-	-	-	-
Narcissistic	0.000	0.000	0.000	0.000	0.000	0.000	-	-	-	-	-	-	-
Borderline	0.154	0.376	0.294	0.588	1.158	0.834	16.576	.002**	.305	0.2332	**.0007*** **	1.000	a<c
Antisocial	0.000	0.000	0.000	0.000	0.053	0.229	1.612	0.447	-.009	-	-	-	-
Negative Symptoms	0.167	0.389	0.647	0.493	0.824	0.393	8.398	**<.001*** **	.243	**.031***	**.001*** **	0.904	a<b; a<c
CGI-S	2.950	0.826	4.250	1.293	4.650	0.745	16.300	**<.001*** **	.338	**.003** **	**<.001*** **	0.491	a<b; a<c
CGAS	60.900	11.867	56.350	11.695	49.450	8.550	5.686	**.006** **	.135	0.565	**.004** **	0.144	a>c
SOFAS	60.900	11.867	59.500	11.109	50.900	9.341	5.005	**.010** **	.118	1.000	**.015** **	**.045***	a>c; b>c

Significance: * = *p*<.05; **=*p*<.01; **=*p*<.001

Note: [a] only administered to patients from 14 y.o. on. Groups: a=headaches; b=REDs; c=NSSIs

Abbreviations: ADHD: Attention Deficit Hyperactivity Disorder; AN: anorexia nervosa; CGAS: Children's Global Assessment Scale; CGI-S: Clinical Global Impression-Severity; GAD: generalized anxiety disorder; OCD: obsessive-compulsive disorder; ODD: oppositional defiant disorder; PRI: perceptual reasoning index; PSI: processing speed index; PTSD: post-traumatic stress disorder; SOFAS: Social and Occupational Functioning Assessment Scale; VCI: verbal comprehension index; WMI: working memory index.
