# Supplementary material for: Look at My Body: It Tells of Suffering—Understanding Psychiatric Pathology in Patients Who Suffer from Headaches, Restrictive Eating Disorders, or Non-Suicidal Self-Injuries (NSSIs)
Source: Pediatr Rep. 2025 Feb 8;17(1):21. doi: 10.3390/pediatric17010021 (PMC11858191; doi:10.3390/pediatric17010021)
Supplement: Supplementary file 1 [file pediatrrep-17-00021-s001.zip › Table S3.pdf]

Table S3. Comparisons between the R-PAS variables in the three groups and Bonferroni post hoc analyses.

	**Headaches** *N*=20		**REDs** *N*=20		**NSSIs** *N*=20					**a—b**	**a—c**	**b—c**	**Contrast**
	M	*SD*	*M*	*SD*	*M*	*SD*	F	*p*	ω^2	*p*	*p*	*p*	
	**Page 1**												
	Administration. Behaviors and Observation												
Pr	101.35	13.23	99.00	10.88	102.10	13.80	0.535	0.765	-.023	-	-	-	-
Pu	108.05	16.810	109.05	14.877	99.90	9.673	5.066	0.079	.049	-	-	-	-
CT	103.95	15.212	107.75	14.935	105.65	15.722	0.299	0.861	-.024	-	-	-	-
	Engagement and Cognitive Processing												
Complexity	101.45	17.617	104.85	10.132	101.05	15.859	0.394	0.676	-.021	-	-	-	-
R	104.80	13.532	111.45	13.485	104.00	14.524	1.743	0.184	.024	-	-	-	-
F%	107.10	1.689	97.75	13.094	99.95	13.209	2.971	0.06	.062	-	-	-	-
Blend	99.35	11.78	99.90	11.276	106.15	8.356	4.7717	0.092	.049	-	-	-	-
Sy	100.90	11.411	98.25	11.920	103.25	10.477	2.328	0.312	-.001	-	-	-	-
MC	98.85	10.054	100.90	12.603	104.30	14.209	0.984	0.38	-.001	-	-	-	-
MC-PPD	102.65	11.32	95.75	16.955	103.40	10.99	1.989	0.146	.032	-	-	-	-
M	99.75	12.611	101.30	12.482	103.70	13.842	1.449	0.485	-.018	-	-	-	-
M/MC	102.471	15.871	100.647	14.722	102.250	10.363	0.086	0.917	-.038	-	-	-	-
(CF+C)/SumC	96.700	13.013	104.181	12.432	104.889	16.058	1.073	0.356	.005	-	-	-	-
	Perception and Thinking Problems												
EII-3	109.55	14.720	120.05	19.289	121.35	22.255	3.608	0.165	.042	-	-	-	-
TP-Comp	111.20	13.281	116.65	17.602	118.40	19.602	1.5132	0.4693	-.001	-	-	-	-
WSumCog	99.90	10.498	103.05	15.14	108.50	15.025	4.459	0.108	.033	-	-	-	-
SevCog	100.25	11.863	102.30	14.143	107.45	13.705	4.661	0.097	.018	-	-	-	-
FQ-%	113.90	16.322	120.80	17.952	119.75	17.779	0.917	0.406	-.003	-	-	-	-
WD-%	111.75	15.117	112.40	16.615	113.50	19.321	0.053	0.948	-.033	-	-	-	-
FQo%	92.90	13.018	83.80	13.181	83.55	15.23	2.961	0.06	.061	-	-	-	-
P	98.3	13.405	92.1	13.665	94.9	9.978	1.241	0.297	.008	-	-	-	-
	Stress and Distress												
YTVC'	98.35	13.232	106.60	17.981	102.80	13.205	2.905	0.234	.017	-	-	-	-
m	102.65	13.539	104.84	11.062	100.20	8.244	2.089	0.352	-.005	-	-	-	-
Y	102.35	14.901	108.10	14.212	110.65	11.348	4.848	0.089	.031	-	-	-	-

MOR	104.55	12.416	103.60	13.012	109.40	13.766	2.013	0.366	.004	-	-	-	-
SC-Comp	91.36	8.732	100.92	15.163	101.86	14.501	4.233	0.1205	.060	-	-	-	-
	Self and Other Representation												
ODL%	98.75	13.314	92.70	14.499	96.90	12.091	3.257	0.196	.003	-	-	-	-
SR	101.85	9.842	100.70	13.055	103.85	14.908	0.458	0.795	-.023	-	-	-	-
MAP/MAHP	131.00	NA	123.00	24.042	121.67	15.885	0.092	0.914	-.434	-	-	-	-
PHR/GPHR	97.11	13.363	112.11	16.069	110.29	16.702	5.098	**.009****	.132	**.014***	**.044***	1.000	a<b; a<c
M-	105.25	11.841	110.05	12.34	111.65	17.184	2.609	0.271	.004	-	-	-	-
AGC	105.95	16.612	97.10	13.784	99.45	12.348	2.039	0.14	.033	-	-	-	-
H	104.65	12.309	97.95	14.398	105.65	9.287	2.969	0.227	.043	-	-	-	-
COP	104.20	10.019	101.30	9.240	108.05	12.352	4.359	0.113	.033	-	-	-	-
MAH	103.85	11.403	98.55	7.149	101.45	10.298	2.627	0.269	.015	-	-	-	-
	**Page 2**												
	Engagement and Cognitive Processing												
W%	104.05	13.617	92.85	10.791	95.55	10.971	4.855	**.011***	.114	**.013***	0.082	1.000	a>b
Dd%	93.0	12.574	112.8	10.294	105.8	11.077	15.64	**<.001*****	.328	**<.001*****	**.002****	0.168	a<b; a<c
SI	103.15	12.918	105.05	13.625	97.75	14.308	3.379	0.185	.018	-	-	-	-
IntCont	99.75	8.961	99.45	11.87	104.30	13.043	1.631	0.442	.004	-	-	-	-
Vg %	99.10	6.373	101.75	7.986	103.15	8.506	2.871	0.238	.014	-	-	-	-
V	98.15	6.192	99.55	7.830	101.60	8.312	3.376	0.185	.002	-	-	-	-
FD	99.20	9.534	103.15	9.544	107.65	12.001	4.383	0.112	.071	-	-	-	-
R8910%	99.30	11.319	98.85	9.444	99.35	10.122	0.014	0.986	-.034	-	-	-	-
WSumC	99.1	12.139	103.6	13.260	104.1	12.38	1.544	0.462	-.002	-	-	-	-
C	103.60	10.679	103.85	11.061	104.90	11.271	0.178	0.915	-.032	-	-	-	-
Mp/(Ma+Mp)	87.50	8.33	101.00	17.756	93.75	16.232	4.012	0.135	.067	-	-	-	-
	Perception and Thinking Problems												
FQu%	98.00	12.974	101.55	16.353	102.50	18.486	0.712	0.700	-.019	-	-	-	-
	Stress and Distress												
PPD	95.80	14.156	103.05	15.86	102.80	7.702	3.868	0.145	.032	-	-	-	-
CBlend	100.7	7.881	98.7	7.072	105.4	11.137	8.318	**.016***	.063	0.688	0.284	**.012***	b<c
C'	102.50	10.060	108.10	18.166	96.35	9.996	6.633	**.036***	.088	1.000	0.267	**.035***	b>c
CritCont%	103.15	10.835	102.55	12.568	112.00	16.18	3.127	0.051	.066	-	-	-	-

	Self and Other Representation												
SumH	104.30	13.546	103.55	11.901	100.15	10.801	0.664	0.519	-.011	-	-	-	-
NPH/SumH	96.50	15.572	108.42	19.001	93.00	13.711	4.475	**.016***	.114	0.093	1.000	**.020***	b>c
V-Comp	104.40	15.836	102.65	11.310	97.95	10.753	1.35	0.267	.012	-	-	-	-
r	111.50	16.873	105.15	12.795	107.90	16.023	2.210	0.331	-.005	-	-	-	-
p/ (a+p)	92.82	13.111	98.38	17.557	95.78	15.799	0.526	0.594	-.019	-	-	-	-
AGM	102.25	12.043	100.00	9.487	106.10	15.420	1.763	0.414	.007	-	-	-	-
T	97.25	6.544	104.10	11.947	100.20	9.03	2.128	0.345	.052	-	-	-	-
PER	101.65	14.365	100.00	8.944	111.30	15.865	8.203	**.017***	.095	1.000	**.047***	**.033***	a<c; b<c
An	107.25	9.673	111.70	15.954	112.60	11.578	1.517	0.468	.001	-	-	-	-

Significance: * = *p*<.05; **=*p*<.01; ***=*p*<.001

Groups: a=headaches; b=REDs; c=NSSIs.

Note: see Table S4 for the legends of all R-PAS variables.
