# Supplementary material for: Look at My Body: It Tells of Suffering—Understanding Psychiatric Pathology in Patients Who Suffer from Headaches, Restrictive Eating Disorders, or Non-Suicidal Self-Injuries (NSSIs)
Source: Pediatr Rep. 2025 Feb 8;17(1):21. doi: 10.3390/pediatric17010021 (PMC11858191; doi:10.3390/pediatric17010021)
Supplement: Supplementary file 1 [file pediatrrep-17-00021-s001.zip › Table S4 RPAS variables legend.pdf]

Table S4. Legend of all R-PAS variables

Pr SS	Prompt Standard Score
Pu SS	Pull Standard Score
CT SS	Card Turning Standard Score
Complex SS	Complexity Standard Score
R SS	Responses Standard Score
F% SS	Simplicity Standard Score
Blend SS	Blend Standard Score
Sy SS	Synthesis Standard Score
MC SS	Human movement and the weighted sum of color determinants Standard Score
MC-PPD SS	MC - potentially problematic determinants Standard Score
M SS	Human Movement Standard Score
M/MC SS	Proportion of Human movement and Human movement and the weighted sum of color determinants Standard Score
(CF+C)/SumC SS	Proportion of with Color and Form and only Color responses Standard Score
EII-3 SS	Ego Impairment Index- 3 Standard Score
TP-Comp SS	Thought & Perception Composite Standard Score
W SumCog SS	Weight Sum of Cognitive Coding Standard Score
SevCog SS	Weight sum of Severe Cognitive Coding Standard Score
FQ-% SS	Form Quality minus Standard Score
WD-% SS	Whole or Detailed response with form quality minus Standard Score
FQo% SS	Form Quality Ordinary Standard Score
P SS	Popular responses Standard Score
YTVC' SS	Sum of light-dark and achromatic determinants Standard Score
m SS	Inanimate movement Standard Score
Y SS	Light-dark responses Standard Score
MOR SS	Deteriorated content Standard Score
SC-Comp SS	Suicide Concern Composite Standard Score
ODL% SS	Oral Dependency Language Standard Score
SR SS	Space reversal Standard Score
MAP/MAHP SS	Proportion of Responses with Mutual of Autonomy Pathological Standard Score
PHR/GPHR SS	Proportion of Poor or Good Human Representation Standard Score
M- SS	Human movements with form quality minus Standard Score
AGC SS	Aggressive contents Standard Score
H SS	Human Content Standard Score
COP SS	Cooperative responses Standard Score
MAH SS	Mutual of Autonomy Healthy Standard Score
W% SS	Whole Standard Score
Dd % SS	Unusual Detail Standard Score
SI SS	Space Integration Standard Score
IntCont SS	Intellectualized Content Standard Score
Vg % SS	Vague Standard Score
V SS	View Standard Score
FD SS	Dimension form Standard Score
R8910% SS	Proportion of responses at tables VIII, IX, X Standard Score
WSumC SS	Weighted sum of color determinants Standard Score
C SS	Color Standard Score

Mp/(Ma+Mp) SS	Proportion of passive Human movement Standard Score
FQu% SS	Form Quality unusual Standard Score
PPD SS	Potentially problematic determinants Standard Score
Cblend SS	Sum of the blend of achromatic/light dark and color determinants Standard Score
C' SS	Achromatic color Standard Score
CritCont% SS	Critical Contents Standard Score
SumH SS	Sum of Human Content Standard Score
NPH/SumH SS	Proportion of not pure H responses Standard Score
V-Comp SS	Vigilance Composite Standard Score
r SS	Reflections Standard Score
p/(a+p) SS	Proportion of passive Movement Standard Score
AGM SS	Aggressive Movement Standard Score
T SS	Texture Standard Score
PER SS	Personal responses Standard Score
An SS	Anatomy Standard Score
